# The Role of Sports Practice in Young Adolescent Development of Moral Competence

**DOI:** 10.3390/ijerph17155324

**Published:** 2020-07-24

**Authors:** Małgorzata Bronikowska, Agata Korcz, Michał Bronikowski

**Affiliations:** 1 Department of Recreation, Poznan University of Physical Education, 61-871 Poznań, Poland; 2 Department of Didactics of Physical Activity, Poznan University of Physical Education, 61-871 Poznań, Poland; korcz@awf.poznan.pl (A.K.); bronikowski@awf.poznan.pl (M.B.)

**Keywords:** adolescents, amateur/professional sport, individual/team sport, moral competence assessment, moral development evaluation

## Abstract

The study examined the moral competence levels in adolescents participating in individual/team sports compared with those not undertaking sports at all. In total, 827 students aged 15–17 years old (45.4% boys, 54.6% girls) from randomly selected secondary schools in the Wielkopolska region in Poland participated in the study. The moral competences were assessed using the Lind’s Moral Competence Test. The students also answered questions concerning their statues of involvement in sport (not involved; amateur; professional), years of involvement, and the type of sport they partook in (individual/team). The results highlight that the moral competence level in most of the examined adolescents (71.6% girls, 76.8% boys) was low. Those who presented a high moral competence level were 10.4% in girls, 8% in boys. There was no interaction between modes of involvement in sport and moral competence when comparing adolescents. The moral competence levels were not correlated with years of training in either mode of involvement or with type of sports. Therefore, we assume that, at this age, the type of sport and the level of engagement do not differentiate moral competence level and that there must be more factors contributing to this. This opens new directions for further research on the role of external factors stimulating the socio-moral growth of youth.

## 1. Introduction

It is quite early in our upbringing that we are introduced to certain standards of behavior, primarily via simple observation, later by noticing rules being applied in certain situations by significant others (grown-ups), and after that by being educated to follow those self-same rules and social conventions. Naturally, context can influence one’s moral behavior. For example, poor child–parent interactions in the early years and living in a poor neighborhood are predictors of moral disengagement in adolescent years [1]. Relatedly, exposure to violent video games is associated with higher level of moral disengagement, leading to worse self-control and higher levels of cheating and aggression in youth [2].

Universal values and cultural traditions are a common bond for overall patterns of coexistence and interaction—in school classes, local communities, religion, or sport groups—at least in theory [3]. However, we are aware that reality has more varied and diverse ways of influencing one’s personal growth, specifically in terms of moral development. Certain (moral) behaviors maybe an outcome of individual decisions based on a person’s maturity of moral reasoning and judgement competency or/and situational contexts, with embedded rules or principles. 

In terms of education, there is also the question of teaching/coaching style. In the case of sport, Hodge and Lonsdale [4] report that coaches who tend to supervise athletes in a very controlled way elicit a higher level of moral disengagement, and increasing antisocial behaviors towards opponents and teammates alike, while, conversely a supportive coaching style precipitates the opposite behaviors of the young athletes. While the research findings of Theodoulides [5] indicate that some physical education (PE) teachers tent to reward the fittest pupils, and in their teaching perform only instrumental actions that emphasize behaviors aimed at winning, sometimes allowing for behaviors leading to unsporting/unfair play in the process. Other studies [6,7] found that performance-oriented goals are associated with several moral variables, such as the legitimacy of injurious acts, approval of unsportsmanlike play, verbal/physical aggression, and antisocial judgments and behaviors. Elsewhere, Stoll [8] observed that participation in competitive sport may sometimes cause disruption of the proper moral development of young people. A facilitative context may play an amplifying role in unethical behaviors and high dispositional moral disengagement in ethically problematic circumstances [9]. However, this can be counteracted with specially designed educational interventions [10,11].

Moral dilemmas containing specific tasks, appropriately designed and carefully interwoven in the process of PE and sport (PES), enhance pupils sound development. The school PES environment is one setting that might be challenging for a young person and can help create a valuable context for experiencing moral decision-making processes. However, Bustamante and Chaux [12] report that interventions containing elements of critical thinking reduce the level of moral disengagement in adolescents. Other research shows that, when combined with PE moral education programs bring positive changes in prosocial behaviors in sport-related contexts [13]. Another study indicates the potential benefits of involvement in sport, specifically with socially vulnerable youth to make them less vulnerable [14]. When such training in teaching values is provided to the teachers, it may help in facilitating both teachers’ and students’ reflection and transfer of values beyond PES [15].

Shields and Bredemeier [16] argue that participation in physical activity (PA) positively influences character development, but it might, in certain conditions, be disruptive in one’s moral growth. They report findings from research [16] on college athletes, among whom those with more mature moral reasoning appeared to be less approving of aggressive tactics than those with less mature reasoning. This was also noticed by coaches, who observed that athletes with “principled” moral reasoning scores were rated as significantly less aggressive [17]. Research shows that some psychological characteristics like aggression, especially in sport, can also be associated with genetic factors influencing sport performance [18], with retaliatory relational aggression [19], or with fatigue that triggers loss of self-control, specifically in young individuals with high moral disengagement [20]. However, can this explain the whole range of pro- or antisocial behaviors in youth sports? 

According to the research findings, the problem is still valid despite the decades that have passed and findings are inconclusive. This proves that further research into this phenomenon is essential.

PES provide a platform for building prosocial appropriate behaviors during the most formative years of a person’s moral development. Each PES session creates countless opportunities for cooperation, decision-making, rule abiding, and fair play behaviors at various levels of competition, all within a climate of social responsibilities and sportsmanship. All these should enhance the development of moral competences. More specifically, when tasks are adjusted to the developmental period, they become valuable tools in stimulation of moral growth of youth. 

However, this is not always the case. Beller and Stoll [8] report that in some circumstances, the behavior of college athletes actually worsens, particularly when there is too much emphasis on a “win-at-all costs” attitude. However, this largely dependents upon the motivational climate created by a PE teacher/sport coach during the training session, and thus their moral competence level (MCL) is also an issue. Henkel and Eearls [21] found that these agents of a child’s moral development are, on average, less developed in their moral reasoning capacities than most of their peers with a similar educational background and age. The latest research by Bronikowska and Korcz [22] indicates a problem—78% of PE major students at university were classified as having low MCL themselves. Interestingly, the increased number of years of professional training in a group of 19-year-old recruits of PES majors was associated with a higher MCL, specifically in male students [23]. Furthermore, Cummings et al. [24] indicate that it is a wider problem—MCLs of preservice and in-service teachers are generally relatively low. The importance of highlighting the moral aspects in training for the teaching profession and in teacher education programs has been raised in other studies [25,26]. Vella, Oades, and Crowe [27] signaled that even if sport coaches have access to formal coach training, coach education programs lack content that is relevant to positive youth development, instead maintaining a focus on technical and tactical skills.

An ambiguous question can be asked as to whether a given type of sport can play a crucial role in stimulating moral growth as, for example, team sports provide quite a different sporting context of experiences than individual sports. While comparing personality characteristics of athletes in team sports (TS) and individual sports (IS), Nia and Besharat [28] found that athletes from TS scored higher on agreeableness and sociotropy, whereas representatives of IS scored higher on conscientiousness and autonomy scales. According to these authors, through individual management and control in IS, one learns order and discipline and builds conscientiousness to rely on oneself, while in TS, the final outcome never depends on the performance of just one player, and unpredicted interferences of many team members mediate many contextual behaviors. These findings confirmed the earlier ones of Eyseneck et al. [29] on the possible psychological differences between IS and TS competitors. There is also a difference in the kind of effort—TS require working together toward common goals in a team, while in IS it is instead a case of working toward one’s own goals. The situation regarding responsibility for the result is also different (shared by many in TS vs borne solely by one person in IS). The effects of involvement in various types of sports is reflected in terms of developing different social skills, with cooperation, communication, coping with pressure, support, and responsibility, which dovetails with one’s intellectual and emotional development and can also play a role in the overall development of MCL.

For the theoretical framework of our research, we used the model of Kohlberg’s moral development [30] in its neo-Kohlbergian version [31]. Kohlberg believed that it is the setting and context that influence moral development and affect the experiences of an individual. In his works, the three-level model [30] consisted of a sequence of 6 stages of moral development. An individual develops morally from the pre-conventional, through conventional, to postconventional level (only a very few reach the highest stages of moral development in their entire lifetimes). Kohlberg also noticed some cases of young individuals who regressed from ostensibly principled moral judgement in their last years of high school to lower scores in their final years of college [30]. By contrast, Rest found [32] that, generally, moral development increases until early adulthood and may plateau thereafter. Later research of Rest et al. [33] showed that, in terms of developing moral reasoning competences, it is personal interest that is first developed in childhood. This is followed by maintaining the norms schema during the adolescent stage, with the development of post-conventional schema emerging while entering adulthood. The latter is the highest schema of critical evaluation of laws, human rights and social norms, decision making, and behaviors based on universal moral principles. Ideally, this should be the outcome and effect of school education, including PES, and should play a critical role in the moral growth of a young person. 

Modern sport faces challenging issues—doping, cheating, bribery, match-fixing, and aggression—that push youth (often via resistance of their parents) away from sport or causes an increase in drop-out rates. Discovering the most critical periods for the development of moral virtues in children and youth, and relating it to the forms of sports may help discover the relationships and help counteract these negative outcomes before it is too late. For that reason, we posed the following research question: Does participation in sporting activities modify the level of moral competence in young adolescents? Therefore, based on the abovementioned findings and theoretical propositions, we developed a cross-sectional study, which aimed at assessing MCL in young adolescents participating in individual and team sports, and compared with those not participating in any sport at all. 

## 2. Methods

Research was carried out in 2018 and included 827 students from several randomly selected secondary urban schools in the Wielkopolska (Eng. Greater Poland) region in Poland (45.4% boys, 54.6% girls aged 16.5 ± 0.6 years). Participation in the survey was voluntary, and the total return rate was 89%. Questionnaires were completed in whole-class groups during a regular school lesson in quiet classroom conditions and took approximately 30 min to complete.

### 2.1. Data Analysis

Due to the lack of normal distribution, comparative analysis of differences between the groups was undertaken with the use of the U Mann–Whitney test. To analyze the potential role of involvement in sport (years of training) on MCL in male and female students a Kruskal–Wallis non-parametric analysis of variance was used. It was also examined whether, and to what extent years of training and moral competence were correlated in each of the groups (training mode: amateur/professional; type of sport: IS/TS) of boys and girls. To test the relationships between variables, a simple Spearman correlation test was used, with the value of correlation strength: ≤0.39 weak, 0.40–0.59 moderate, and ≥0.60 strong [34]. Significance was set at *p* ≤ 0.05. Statistical analysis was carried out using Statistica 13.0 software (StatSoft, Krakow, Poland).

### 2.2. Research Tools

To measure MCL, Lind’s Moral Competence Test was used [35]. To this study, a validated and certified version was used [36]. Participants were requested to confront two moral dilemmas and agree or disagree with the statements which were presented to them. One concerned facing a situation of illegal behavior at work, another was a life-saving dilemma. Students responded on a nine-point Likert-type scale, from −4 (totally disagree) to +4 (totally agree). Each story had 12 statements (six in favor and six against the proposed behavior). All statements corresponded to one of six stages of moral development [30]. The summarized score, called the *C-Index*, is computed and ranges from 1 to 100. It actually measures a person’s ability to assess an argument based on their moral quality or, in simpler terms, the degree to which a person allows their personal judgments to be affected by moral concerns or principals rather than their personal opinions and constructions. *C-index* scores below 9 are considered to be an extremely low, scores 10 to 19 are considered low, scores 20 to 29 are considered medium, 30 to 39 are considered high, 40 to 49 very high, and above 50 extremely high. 

In addition, the examined students were asked to answer question concerning their statues of involvement in sport (no involvement, amateur, and professional). Professional participation meant engagement in regular system of competitions, organized by a sports federation, whereas amateurism meant taking part in sports as a hobby, for pleasure [37]. Then, they were asked to declare the number of years of involvement and the type of sport they partook in. This was later categorized by the research group into either a IS and TS based on the following criteria: TSs concerned activities with common efforts of one team playing against another team, which included cooperation, interaction and shared responsibilities; whereas ISs were those where the final result and responsibilities depend on the efforts of an individual and which is usually played in a one-on-one scenario. 

### 2.3. Ethics 

The investigation was carried out following the rules of the Declaration of Helsinki of 1975 and revised in 2013. The study protocol was approved by the Local Bioethics Committee of University of Medical Sciences in Poznań (decision no. 893/18) before undertaking the research.

For the students’ convenience, the information about the anonymous and voluntary nature of their participation were read out before completing the questionnaire, that the study records would be kept confidential, and that their individual contributions would be unidentifiable in the final report. Written consent was collected from all the participant and their legal careers. 

## 3. Results 

In the examined group (*N* = 827), there were 139 students (43.1% boys, 56.9% girls) who declared no participation in any form of sports; 462 (66% boys, 34% girls) declared involvement on an amateur level; and 226 (51% boys, 49% girls) were involved in sport within professionally organized structures. Among those who declared an amateur level of involvement in sport, 306 (36.3% boys, 63.7% girls) related to IS and 156 (61.5% boys, 38.5% girls) with TS. Among students engaged professionally, 116 (37.9% boys, 62.1% girls) trained in IS and 110 (58.1% boys, 41.9% girls) in TS. There were no statistically significant differences (U Mann–Whitney test) between the groups in terms of the *C-index* of moral competences, which was established for the “no sport” group at a mean value of 15.1 ± 11.9, for the amateur group at 14.2 ± 9.6, and for the professional group at 14.0 ± 9.8. A U Mann–Whitney test indicated there were also no significant differences noticed between boys and girls. 

The first step was to determine whether there was any relationship between the MCL among students taking no part in sport, those practicing in at an amateur level, and those who trained in a professional mode. ANOVA Kruskal–Wallis testing for different modes of sport involvement as an independent variable showed no significant differences between the examined groups (no sport, amateur, professional) and MCL (*H* (2, *N* = 827) = 0.15159; *p* = 0.927).

The second step was to investigate whether involvement in IS or TS made a difference for boys and girls in terms of MCL. The U Mann–Whitney test showed no statistical differences between the two forms of sport participation (IS/TS) in terms of the MCL between boys and girls, but a more detailed examination showed some differences (*p* = 0.012) between male and female students professionally training TS, with boys scoring lower than girls (Table 1).

**Figure 1:**
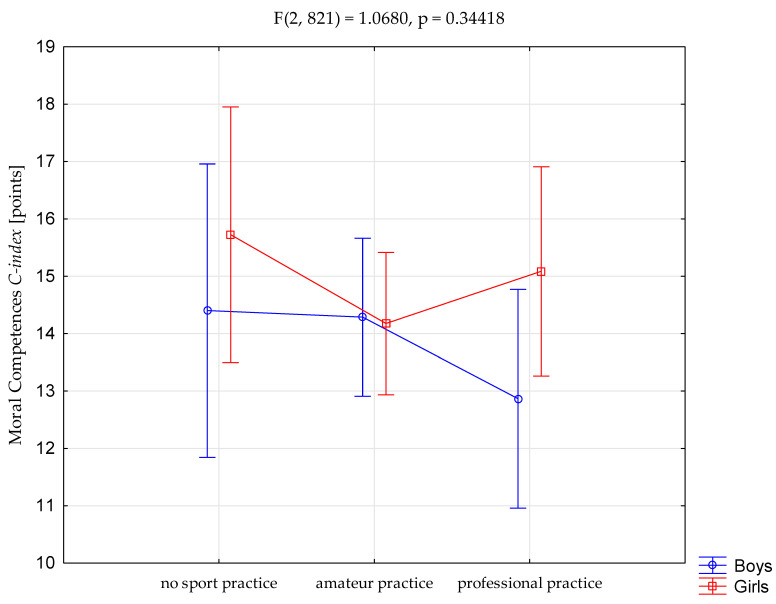
Two way interaction between various modes of involvement in sports and the level of moral competences in boys and girls.

Next step was to see whether the differences would occur in modes of sport involvement x gender analysis. For that reason a Kruskal–Wallis test (ANOVA) was used and indicated no statistically significant differentiation (*F*(2, 821) = 1.068; *p* = 0.344) between boys and girls (Figure 1). 

This was followed by an analysis of correlations of examined variables (Table 2). There were no statistically significant correlations found. Number of years of training did not correlate with the MCL in any type of sports (IS/TS), nor with the mode of sport involvement (amateur/professional) either in boys or girls. We also checked the whole sample for the potential correlation of MCL with age (the cohort included 15, 16, and 17-year-old students), but there was also no correlation found (*r* = 0.01; t(N-2) = 0.199; *p* = 0.841).

## 4. Discussion 

The above study aims to portray an integrated description of involvement in sports and its potential relationship to MCL among adolescents. Research shows there is no interaction between modes of involvement in sport and MCL when comparing adolescents; this concerns both boys and girls. MCL is not correlated with number of years of training in either mode of involvement (amateur/professional) nor type of sports (IS/TS). Likewise, no correlation of MCL with age is noticed. The examined adolescent boys and girls presented similar MCL irrespective of whether they trained professionally or just recreationally as amateurs. The only significantly lower MCL is noticed in boys practicing TS in a professionalized way when compared to girls. We can assume that at the age between 15–17 years, the type of sport and the level of engagement do not differentiate the MCL and that there must be more factors contributing to this. However, the most interesting observation is the MCL itself (*C-index* around 15 points average for the whole cohort), which, in a majority of the examined youth (74% of the examined sample; 71.6% girls and 76.8% boys), is low (below 19 points), whereas the number of those who presented a high MCL (*C-index* above 29 points) is only 10.4% in girls and just 8% in boys. It seems that the findings reflect natural rate and state of development of moral competence in young adolescents. The findings are in line with observation of other authors [38,39,40]. Power and Higgins [40] found in their study that there was significant (but still modest) growth in moral reasoning and improvements in moral behavior in young adolescents. Moreover, Bronikowski [41] explains that young adolescents need sense of coherence (of understanding) to maintain their engagement in physical activity, but also to pay attention to the moral standards that are often neglected by PE teachers and thus are not considered as important in sports. This kind of involvement has limited influence on their social and moral development. Therefore, it seems that specially designed school interventions could prove effective in keeping desirable yet sustainable moral development patterns throughout the life span if they could only become meaningful for the youth [40]. Previous research [42] strongly indicates also the importance of the perceived moral atmosphere, which is linked to lower incidents of adolescent misbehavior and higher incidence of prosocial behavior observed for example during sports activities.

The PES setting is considered to be legitimate environment for the development of child and youth moral norms and attitudes [43], though it should not be the only context for such efforts. Behaviors presented by youth in educational settings can be stimulated, monitored, and corrected by a qualified educator, in order to develop the desired levels of awareness of moral standards because there is a social imperative for PE to teach life skills essential to functioning in everyday life outside of the sport setting [44]. This is what traditionalists would say, referring back to the times of Plato and the Greek *arête* or to more recent times of Arnold’s philosophy of moral and pedagogical education at Rugby school [45,46,47,48]. Just a 100 years ago de Coubertin, built his idea of neo-Olympism around the ancient *kalokagathos* (English: “beautiful” and “good” or “virtuous”), which he saw as the most universal moral values. At that time, it was still socially expected that youth presented universally accepted virtues that referred to the socially accepted moral standards (i.e., the *religio athletae* concept) [49]. To a great extent, little has changed in the last century—the same objectives of moral education are still present and well-defined in most of PE curricula around the world [50,51]. How is it that the outcome of schooling and sporting experience (in terms of moral competence) remains so low? 

School education should be the most critical period for personal growth, in various dimensions. The social perception that moral competence could be and should be promoted via PE and school sport was a dominant theory till the turn of the 20th century [52,53], with such values like cooperation, fairness, a sense of responsibility, and respect becoming pivotal points for educational tasks. Recently, the questions have been raised though as to whether this could be applicable outside the sports gym, where the sportification of all spheres of life has been observed [54]. Indeed, one may ask whether the problem of low MCL is related to the school environment or an outcome of observed broader social changes with violence, inequality, lack of tolerance and egocentrism in all spheres coming to life more vividly recently [55]. Gini et al. [56] found that, in a school setting, both bullies and defenders show advanced moral competence, integrating information about beliefs and outcomes in judging the moral permissibility of action, while victims show delayed moral competence, focusing on outcome information alone. Interestingly, the bullies, despite their advanced moral competence, were deficient with respect to moral compassion, as least when compared to victims or defenders. It is also possible that there is something about modern sports, even in a school context that does not let youth grow past a basic MCL, and that allows young people for just a temporary engagement in sports. It is possible that “Generation Z” have developed a sense of moral relativism that is much more individualized today than it used to be [57]. Maybe some norms that once used to be the universal base for cultural and moral standards of social life have changed so profoundly that the so-called traditional set of core virtues is not in place anymore? It seems that there are no simple answers here and more in-depth research is needed.

The process of neglecting the traditional set of values has been recently observed more than ever, and one cannot have failed to notice the disturbances in social and cultural relationships at the more general, societal level. Children observe a grown-up world and react accordingly. Feeling like being released from the parental leash, with unlimited access to the “Mighty Internet” and less controlled in their moral conducts and behaviors, has allowed for more individual “experimenting” with moral standards. If the PES environment allows—or even encourages—behavior that neglects the moral standards of traditional values and still work in their personal favor who would not take advantage of such a situation? The outcome is easy to predict: moral values are to be incorporated when suited, and this is how they learn to see it. 

However, one also needs to take into account the factor of ontogenetic developmental principles that might influence the moral maturation process. For example, some teenagers might have a different tempo of transition from one stage of moral reasoning to another or, as we have come to understand through our research, that the process of growing from conventional to nonconventional and to postconventional levels of moral reasoning does not have to be an invariant sequential process, but may, on the contrary, be a complex one, with many factors mediating the change, and might include regression as well [58]. Recently, Garrigan et al. [59] have proposed an integrative framework which combines components of earlier theoretical models of moral development including decision-making findings, contextual factors, and social neuroscience theory. The model shows that growing through moral stages does not need to be sequential, and that individuals are not consistent in their moral reasoning. This opens new alleys for further research on the role of external factors (i.e., sport setting or role of a sport coach) acting as catalysts in stimulating the onward or retrograde moral development of a child. 

The study by Powell et al. [60] indicated that, when we ask children to judge the actions of others, they consider the costs and benefits of harm, similarly to adolescents and adults, but those aged six judge actions involving harm negatively, regardless of the benefits. A younger child may see the opportunity to cheat but rules it out and does not consider it a possible action. This may be due to the basic knowledge or alternatively while experiencing more and more situations and educational support due to the deeper understating of moral necessity, i.e., an appreciation of fairness resulting from their experiences of PES [59].

Opinions on this issue are ambiguous. Shields and Bredemeier [16] believe that PES is a more fertile ground for children’s socio-moral development and character building than competitive sports. However, some [61] argue that the validity and reliability of the research findings of Shields and Bredemeier might be flawed, as the authors struggled to combine two different theories and had problem with conceptualizing it in one integrated yet multifaceted concept of character, which was reduced to primarily cognitive ability of making judgment. Contrary, Carr [62] argues that PES, as with any other school subject, despite involving co-operation, does not qualify as a form of moral education, as there is nothing moral in teaching hockey nor football skills in itself. However, he adds that this does not mean that moral education cannot take place during these classes through, e.g., the cultivation of moral attitudes or development of moral character [62]. There are a lot of situations in PES which require self-critical, sometimes even intuitive thinking in order to resolve the conflicting situations arising in the “heat of the PES action.” Appropriate moral competences and awareness of a sport discipline specific code of behaviors allow youth to select between the option with “keeping the rules” seen as prima facie principle or duty of sports participants [63]. 

On the contrary, it may also become a critical point, when PES teachers/sport coaches’ professionalization culture, or the whole education system in fact, imposes “discursive closure” in terms of morality, gender discourse [64], or lack of skills in critical thinking. It also may be that the problem is related to the MCL or quality of training of PE teachers/sport coaches as role models themselves [22]. Sanderse [65] signifies that role modelling is rarely used as an explicit teaching method, and only a very small percentage of school students consider teachers as role models. According to Chen and Ennis [66], PE teachers present the “disciplinary mastery” orientation focusing on developing performance proficiency in sport skills and understanding of performance-related knowledge. We suggest that more critical thinking skills and moral dilemma tasks need to be included in their training to improve the situation. Therefor there is a huge need to foster social responsibility and to build character as an important quality factor of PES, as participation in sports has the potential to advance moral reasoning skills among its participants [67]. Increasing social demand for life-skills education requires finding the ways of transforming PES combined with health education into a “sustainable development” related subject. Lake et al. (p. 474, [68]) makes a point on that when saying “while active living by definition is concerned with the maintenance of activity behaviours across the life span, the addition of the word ‘sustainable’ serves to emphasize environmental influences on our physical activity behaviours as well as the environmental implications of those behaviours.” Also, studies on Hellison’s model of developing responsibility through physical activity [69,70] provide both evidence and potential pathways of dealing with the matter. Hellison [71] even suggested some strategies (awareness, experience, choice, problem-solving, self-reflection, and counseling time) that could be easily interwoven in the process of PES by a well-trained PE teacher or a sport coach. Also, a study of Šukys et al. [72] confirms that personal role of coaches in moral education encompassing professional knowledge and moral competences of athletes is of great importance. Therefore, in the future, we should investigate such potential or possible covariates that were not explored in this study. 

What is important is the students’ point of view; most of the studies present the researchers’ perspective, ignoring the child’s one. In a study on the moral development of children aged 10–12 with competitive youth sport experience, Stuart [73] revealed that the identified issues concerned three areas—fairness of adult’s actions, negative game behaviors, and negative team behaviors. Examples of each category were unfair actions by coaches, disrespecting opponents, and selfish behavior in practice. She [73] reports in her study that teenagers saw PES as a place where the social-conventional behaviors could be modelled and reinforced, including a variety of issues from outside sport. In a longitudinal study on American youth, Dubois [74] proved that participation in well-organized sport activities positively develops the moral abilities of youth. However, the problem might be the accurate assessment of a child’s readiness for participating in organized, though competitive sports, which sometimes might be the demand of an unqualified coach, or encouragement from more persistent but even less qualified parents, all leading to training misconduct (early specialization and identification of talented players, risky behaviors, injuries, burnout, peer isolation, and moral issues) [75]. Shields and Bredemeier [16] studied the differences between sport students and non-sport students in the context of moral development. Their conclusions of significantly slower rate of moral development of students playing on a college basketball team compared to students whose only activity was limited to participation in PE comes as quite contrary to the prevailing wisdom. However, such an association was not found in the case of older secondary school students. The findings from research [16] also pointed to the correlation between the length (in years) of training in martial arts with significantly slower rate of moral development, which also determined the level of aggression. 

The problem may also be rooted in the contextual factors of the setting. In a study on a very large sample of American students in the educational context, where a low correlation was found between repeated moral behaviors concerning cheating in school tests [76], it was observed that students changed their behaviors from one experiment to another, and it was difficult to recognize any patterns of moral reasoning. So, it may also be the sociological context of each particular situation (different and specific for various sports) that plays a mediating role in triggering certain moral behaviors, which may be altered in the same person in various circumstances. We did not collect data on that issue in this research, which might be considered as a limitation of the study but will serve as a hint for further in-depth research. 

The findings of the current study should be interpreted cautiously because of some design limitations. The size of the sample is certainly a strength of the study, while among the limitations, we can point the cross-sectional design, which indicates that we cannot infer causal relations. Data on moral competence was based on declarations by adolescents and may be subject to some bias. Additionally, no consideration was given for educational (e.g., the moral values of their PES trainers/teachers), sociocultural/socio-economic status (e.g., the opportunity to get involved in sports activities during free time), and environmental (e.g., safe neighborhood) influences that might have affected the moral competency. There is a need to analyze this dependency separately, in detail, and in a multidirectional manner. Longitudinal designs may assist researchers in addressing hypotheses of the level of moral competence in adolescents in relation to their long-time involvement in sport.

## 5. Conclusions

As mentioned above, our detailed examination only shows some differences between students professionally training TS, with boys scoring lower on moral competence than girls. Thus, assuming one can say that, for the 15–17 age group, the type of sport and the level of engagement do not modify the ways it affects moral competence, so there must be more factors contributing to this. This opens new paths for further research in this area on the role of external factors (i.e., sport setting, role of a sport coach in the teaching process, etc.) acting as catalysts for stimulating onward or retrograde moral development. Through the development of society, media, and technology, we cause changes in perception of the societal order of moral values. PE teachers and coaches must understand their role modeling potential and as a matter-of-fact social need to develop instructional methods and delivery teaching styles that will produce effective learning outcomes in the Millennial student in terms of their moral qualities. Future research could take into consideration also moral competence (and gender) of the PES trainers/teachers, as it is possible that the moral competence of the PES trainers/teachers may influence the behaviors of young people.

## Figures and Tables

**Table 1:** Differences (U Mann–Whitney values with mean ± SD) in moral competence *C-index* (points) between adolescent boys and girls in various modes of involvement in sport.

Variables	IS		*p*	TS		*p*
Amateur	Boys (*n* = 155)	Girls (*n* = 160)		Boys (*n* = 167)	Girls (*n* = 106)	
*C-index*	13.5 ± 9.9	14.4 ± 9.3	0.257	15.1 ± 9.3	13.4 ± 10.2	0.102
Professional	Boys (*n* = 44)	Girls (*n* = 72)		Boys (*n* = 64)	Girls (*n* = 46)	
*C-index*	15.1 ± 10.8	14.8 ± 11.0	0.797	11.2 ± 7.6	15.4 ± 9.0	**0.012**

Note. IS—individual sports, TS—team sports. The significant analyses (*p* < 0.05) may be found as bold.

**Table 2:** Spearman correlation between years of training (M±SD) and moral C-index in adolescents.

Type/Mode of Sport Involvement	*N*	*C-index*	Years of Training	R	t(N-2)	*p*
Boys						
IS and amateur training	111	13.5 ± 9.9	4.6 ± 3.2	−0.02	−0.211	0.832
TS and amateur training	96	15.2 ± 9.4	4.5 ± 3.0	0.09	0.972	0.333
IS and professional training	44	15.2 ± 10.8	5.8 ± 2.6	0.26	1.782	0.081
TS and professional training	64	11.4 ± 7.7	6.9 ± 3.1	−0.12	−0.986	0.327
Girls						
IS and amateur training	195	14.4 ± 9.3	5.2±3.6	0.05	0.833	0.405
TS and amateur training	60	13.4 ± 10.2	4.0±2.6	−0.04	−0.310	0.757
IS and professional training	72	14.9 ± 11.0	6.1 ± 3.1	0.02	0.514	0.608
TS and professional training	46	15.4 ± 9.1	5.6 ± 2.4	−0.01	−0.020	0.984

Note: IS—individual sports, TS—team sports.
